# Rules and ward climate in acute psychiatric setting: Comparison of staff and patient perceptions

**DOI:** 10.1111/inm.12980

**Published:** 2022-02-06

**Authors:** Marta Corbetta, Barbara Corso, Carlo Alberto Camuccio

**Affiliations:** ^1^ Self Employed Nurse Padova Italy; ^2^ Neuroscience Institute National Research Council Padova Italy; ^3^ Professor In Charge in Mental Health Nursing University of Padua Padova Italy

**Keywords:** acute care, patient experience, risk management, scales and assessment, staff perceptions

## Abstract

The ward climate or atmosphere refers to its material, emotional and social conditions. A good ward climate in psychiatric settings can influence the mood, behaviour and self‐concept of patients and staff members and improve patient outcomes. Many studies have examined the relationship between ward climate and aggression, but only a few have investigated the effect of a ward’s environment, rules and activities. This multicentric observational study aimed to assess the relationship between the rules/activities and the climate of four acute psychiatric units of Northern Italy. The Essen Climate Evaluation Scheme (EssenCES) questionnaire, which was administered to patients and staff, was used to evaluate the different dimensions of ward atmosphere. There was a good response rate (79%) in patients and staff members who completed the questionnaire (114 patients and 109 staff). Safety perception appeared to be quite different in patients and staff. The patients who were authorized to have more visiting hours and more time to use their mobile phone had higher scores on Experienced Safety subscale. A negative correlation between the Therapeutic Hold and Experienced Safety subscales was found in the staff members, and this was due to their negative perception. The ward climate seemed to be affected by the unit’s rules, especially with respect to visits and the smartphones use. Nurses need to be aware of the importance of ward climate and how their own perception may differ from and that of patients: this gap could lead to decisions detached from the patients’ needs.

## Introduction

The ward climate or atmosphere is a complex concept that is linked to the architectural and organizational features of a facility, the characteristics of its staff and patients, and the relationships between these elements. All of these material, emotional and social conditions are interrelated and overlapping, and their interplay seems to be able to influence the mood, behaviour and self‐concept of both patients and staff members. In psychiatric settings, a good ward climate is often associated with better patient outcomes, a better the therapeutic alliance between the patients and staff and an increased patient satisfaction (Johansson & Eklund, 2004; Middelboe *et al*. 2001; Milsom *et al*. 2014; Moos, 1974; Papoulias *et al*. 2014).

Ward’s rules and daily routines, the unit’s daily organization and activities are constituent and fundamental elements of the ward climate; thus, they can strongly affect how the staff and users perceive it (Alexander & Bowers, 2004; Papoulias *et al*. 2014; Pelto‑Piri *et al*. 2019). Ward routine and psychiatric wards rules do not have a recent and clear definition: they are described by Alexander and Bowers (2004), as a way to limit and control patients' behaviour, for example on smoking, phone calls or use of electronic devices, having time away from the ward, access to different activities and facilities etc.

The Italian psychiatric system has some peculiarities that need to be described because this system could lead to shorter hospitalization, the shortest in Europe (Navarro *et al*. 2021), an important factor influencing the ward climate and the ward rules. In Italy, since 1980, patients needing hospitalization because of an acute psychiatric disorder are admitted to acute units called Psychiatric Diagnosis and Care Services (SPDCs) each with a maximum of 16 beds. According to Navarro et. al (2021) and Dimitri *et al*. (2018), the average length of stay in Italy was 17.9 days. Other high‐income countries as Belgium, England, Germany and USA have, respectively, an average of 55.1, 46.2, 37.0 and 24.9 days. Furthermore, in 2018 in Italy, there were fewer beds for acute care, in proportion with the population’s size, than in the other high‐income countries: if in Italy there were 10.95 beds every 100 000 inhabitants, in England they were 50.63, in Germany 41.08 and in USA 14.36 (Barbui *et al*. 2018).

## Background

According to studies in the literature, a negatively perceived ward climate can impede the construction of a good therapeutic relationship, something that would reduce the impact of the therapeutic environment and patient satisfaction (Schalast *et al*. 2008; Tonkin *et al*. 2012). A relationship exists between adverse social climate and a higher prevalence of inpatient aggression, and this seems to be independent from patients’ diagnoses (Middelboe *et al*. 2001).

Most of the studies on climate have sought to verify how aggression influences the ward atmosphere (McCann *et al*. 2015) and to investigate the strategies that could limit it and thus make the ward safer (Fröhlich *et al*. 2018; Hottinen *et al*. 2020).

The patients and staff of a psychiatric unit can perceive the climate in different ways (BootsMiller *et al*. 1997; Papoulias *et al*. 2014), and, the majority of studies about ward climate, compare patients’ perceptions with those of the staff. This comparison highlights a gap of perception between staff and patients: usually the patients perceive the climate less therapeutic and safer than the staff do. This difference in perceptions needs to be filled, or at least well known, by clinician: for example, the awareness that patients perceive a less therapeutic climate may bring to new therapeutic and care strategies that better meet patient’s needs.

The subject of the rules within psychiatric institutions was addressed by Goffman (1961) in his important work Asylum of 1961, within a broad analysis of the total institutions. His analysis of micro‐relational interactions in psychiatric institutions was particularly influential for psychiatry and sociology in the ‘60s and ‘70s, contributing significantly to the processes of de‐institutionalization. According to some authors, although set in a context completely different from that described by Goffman, his typical ideal analyses of the behaviour of institutions and staff can help us to understand some contemporary processes of psychiatric settings. For instance, Quirk *et al*. (2006) see some risk management practices as attempting to reduce the permeability of the psychiatric ward outwards, as well as McKeown *et al*. (2019) and Adlam *et al*. (2013) that highlight the de facto contradiction between rhetoric of the therapeutic alliance and personalization of care and psychiatric environment increasingly bureaucratic and restrictive.

In recent literature, the topic of ward’s rules is not very developed, although it emerges as one of the central elements of the patients' hospitalization experience (Alexander & Bowers, 2004; Nugteren, *et al*. 2016). It seems clear that ward rules, the type of rules and how they are managed, strongly affect the relationship between patient and nurse and its quality; and there is also clear a link between restrictive rules, loss of autonomy and aggression (Alexander, 2006; Alexander & Bowers, 2004; Nugteren, *et al*. 2016).

Most studies on rules focussed, even in depth, on the effects of the single rules such as open‐door policies, smoking areas, trying to evaluate the influence on specific phenomena like absconding, aggressiveness (Efkemann *et al*. 2019; Lo *et al*. 2018). But literature lack of a specific and comprehensive study on ward rules: if a certain number of studies explore the patients perspective, as evidenced by Nugteren’s *et al*. (2016) systematic review, the nurses perspective seems always oriented to risk management issues (Clancy *et al*. 2014).

Ward climate is measured in psychiatric settings since the ’60s of the last century. In 1968, Moos developed the Ward Atmosphere Scale (WAS), a versatile tool for measuring the social climate by patients and staff; the instrument can also investigate the differences in climate perception between these two groups (Kellam *et al*. 1966; Moos & Houts, 1968; Moos *et al*. 1973).

In 2008, Schalast developed the Essen Climate Evaluation Scheme (EssenCES) questionnaire, which focusses on three specific aspects of the social climate: cohesion, aggression and therapeutic support, factors that are relevant to both the patients and staff in different clinical contexts. The questionnaire has been validated in German (Schalast *et al*. 2008) and has been translated into several languages including Italian (https://www.uni‐due.de/rke‐forensik/essenerstationsklimafragebogenessences.php). The validation studies that have been carried out have confirmed its effectiveness in measuring the perception of the ward climate even from an intercultural point of view (Milsom *et al*. 2014; Tonkin *et al*. 2012), and several research studies have been conducted using it as a measuring tool (De Vries *et al*. 2016; Dickens *et al*. 2014).

This multicentric observational cohort study aimed to investigate the differences in the social climate between patients and staff members of four acute psychiatric units using the Italian version of the EssenCES. Moreover, it intended to explore whether the rules (e.g. use of smartphones), the organizational aspects (e.g. visitors) and the activities of the unit could produce a difference in the social climate.

## Methods

### Study population

The study was conducted at the Acute Psychiatric Units (APU) of four psychiatric facilities located in the Veneto region, a North‐Eastern part of Italy. All the inpatients of the four psychiatric facilities were eligible to participate. The exclusion criteria for the patients were being younger than 18, having stayed less than two days in the facility, a diagnosis of dementia, difficulty in understanding the Italian language, a diagnosis of organic psychosis, acute unstable phase of the psychiatric condition not allowing them to fill out the questionnaire as indicated by the staff, and unwilling to participate in the study. All of the staff members who were regularly employed at one of the units were eligible.

Together with a nurse employed in that specific unit, the first author of the current study provided the staff members and the patients with both verbal and written explanations outlining the study’s aims and methodology and detailing how the data would be collected and stored. The first author also had the responsibility of meeting with each participant to collect their demographic information and to ask them to sign a written informed consent statement, of administering the questionnaires and of answering any questions that arose throughout the various stages of the study. The meeting between the researcher and each individual participant took place in a quiet room.

Between November 2017 and January 2018, a total of 194 patients were hospitalized at one of the four APUs. Thirty‐eight of these patients were excluded since they did not meet the inclusion criteria, leaving 156 eligible subjects. During that time period, 129 staff members were regularly employed in one of the APUs and thus were potential candidates for the study. Seventeen of the patients and 11 of the staff members declined the invitation to participate and 33 subjects (24 patients and 9 staff members) who initially agreed to participate did not turn in the questionnaire. The questionnaire of one patient was excluded because it was incomplete.

### Material and measures

All the participants filled out the Essen Climate Evaluation Schema (EssenCES) questionnaire (Schalast *et al*. 2008), a 17‐item scale which was developed to measure social climate in forensic psychiatric services. The first (‘ice‐breaking’) and last (positively worded concluding) items are not scored. The remaining 15 items are divided equally into three subscales: *Therapeutic Hold* (investigating the extent to which the climate is seen as supportive of the patients’ therapeutic needs); *Patient Cohesion and Mutual Support* (investigating if the mutual support characteristic of therapeutic communities was present); and *Experienced Safety* (investigating the level of perceived tension and threat of aggression or violence). The questionnaire uses a five‐point Likert scale ranging from ‘not at all’ to ‘very much’. Answers’ scores are ranging from 0 to 4 in the *Therapeutic Hold* (except for item 13) and the *Patient Cohesion and Mutual Support* subscales, while from 4 to 0 in the *Experienced Safety* subscale and item 13 of the *Therapeutic Hold* subscale. Higher scores reflect a more positive perception of the various aspects of ward climate investigated by the questionnaire.

The Italian translation of the EssenCES‐IT, which was used here, was validated by the last author of the present work using the translation back‐translation method and a focus group.

Confidentiality and anonymity were ensured since the questionnaires were returned anonymously in sealed envelopes which were inserted into a sealed ballot box positioned in the kitchen unit. After identification codes were generated to ensure anonymity, the participants’ answers were entered into an Excel worksheet.

As far as the patients were concerned, demographic data (i.e. gender and age) and details regarding their current hospitalization such as the discharge diagnosis (psychosis, depression, bipolar disorder, personality disorder, other type), type of admission (voluntary/involuntary admission), length of stay (in days) and the presence of previous hospitalizations (yes or no) were collected from the patients’ medical records or learned from the patients. The contextual variables collected were the room type (single or double/triple room), if the unit allows him/her to spend time away from the ward (yes or no), the smoking status (yes or no), if restraint was needed at the time of hospitalization (yes or no) and if the unit allowed him/her to receive visitors (yes or no).

As far as the staff members were concerned, besides their demographic data (i.e. gender and age) and the highest degree obtained (primary/secondary, BSc/nurse qualification, MSc, specialization or other), they were also asked about their work experience in terms of: professional qualification (psychiatrist, psychologist, nurse, rehab therapist, nursing assistant or other), years spent in Mental Health (MH) services and years spent working at that APU.

The four APUs investigated were classified depending on the following characteristics: the presence of a garden where patients could spend time (yes vs no), availability of organized activities such as psychoeducation and structured activities (yes vs no), visiting hours (10 h/day vs 2 h/day), cell phone hours (2 h/day, 12–13 h/day, 24 h) and location (rural vs urban). The rationale of the choice of these characteristics was based on presence in literature and from the fact that they were general rules usually not dependent on the conditions of the individual or on the discretion of the nurses (i.e. rules as the possibility of having a shower, the possibility of staying in the room in the morning, the access some spaces and rooms). Each of these characteristics was verified in all the APUs by the first author, together with a nurse employed in that specific unit, with a direct field observation.

### Statistical analysis

Quantitative data are presented as mean and standard deviation (SD), while categorical data are reported as absolute numbers and percentages. Since EssenCES total score and one of subscales score were not normally distributed (inspected by Shapiro–Wilk test), non‐parametric tests were applied. The differences, with regard to the ordinal variables, between two groups (e.g. staff vs patients) were assessed by the Mann–Whitney test, the differences between more than two groups (e.g. units) were investigated using the Kruskal–Wallis test, while the Fisher exact test was used for the categorical data. Whenever a significant difference was found between groups, post hoc tests were used to evaluate the differences among specific pairwise contrasts. Effect size was calculated by the Cohen’s *d*. Cohen (1988) suggests that a *d* = 0.2 can be considered a ‘small’ effect size, 0.5 represents a ‘medium’ effect size and 0.8 a ‘large’ effect size. This means that if the difference between two groups’ means is <0.2 SD, the difference is negligible, even if it is statistically significant. Spearman’s Rho was used in correlations.

Factor analysis was conducted in order to evaluate whether the three‐factor structure of the original version was retained. The internal consistency was assessed using Cronbach's alpha (α) and corrected item total correlation (CITC) coefficients. To be adequate, the α needed to exceed 0.70, while a CITC of 0.50 was considered high and items with a CITC value less than 0.20 were removed. Internal consistency was examined for the population sample as a whole, as well as for the staff and patients separately.

Data analysis was carried out using STATA‐SE v15.1 (StataCorp, College Station, Texas).

The study was carried out following the STROBE guidelines (von Elm *et al*. 2008).

### Ethical issues

Formal approval of the study was obtained from the Italian Health Authorities and the Local Departments of Mental Health. Confidentiality and anonymity were guaranteed and treated as dictated by Italian law, and the participants were informed that they could withdraw from the study at any time. The patients expressed their willingness to participate by signing a formal written informed consent.

Before the data were made available to the authors, patients’ identifiers were replaced with anonymous numerical codes making it impossible to identify the individuals involved. Given the stringency of this procedure, Health Authorities and Mental Health Departments considered that it was not necessary to seek authorization from the local ethics committee.

The study was carried out in accordance with the principles of the Declaration of Helsinki.

## Results

### General characteristics of the sample

Descriptive statistics of the patients and the staff employed in each unit and for the total sample are outlined in Tables 1 and 2.

**Table 1:** Descriptive statistics of patients by unit

Unit	A (N = 28) (%)	B (N = 32) (%)	C (N = 34) (%)	D (N = 20) (%)	Total (N = 114) (%)	*P*‐value
Gender						
Male	15 (55.6)	18 (56.3)	18 (52.9)	15 (75.0)	66 (58.4)	0.425
Female	12 (44.4)	14 (43.8)	16 (47.1)	5 (25.0)	47 (41.6)	
Age class						
18–20	0 (0.0)	1 (3.1)	2 (5.9)	0 (0.0)	3 (2.6)	0.148
21–30	0 (0.0)	4 (12.5)	5 (14.7)	2 (10.0)	11 (9.6)	
31–40	2 (7.1)	4 (12.5)	7 (20.6)	3 (15.0)	16 (14.0)	
41–50	3 (10.7)	10 (31.3)	6 (17.6)	6 (30.0)	25 (21.9)	
51–60	11 (39.3)	5 (15.6)	7 (20.6)	6 (30.0)	29 (25.4)	
61–70	9 (32.1)	3 (9.4)	5 (14.7)	2 (10.0)	19 (16.7)	
70+	3 (10.7)	5 (15.6)	2 (5.9)	1 (5.0)	11 (9.6)	
Diagnosis						
Psychosis	8 (28.6)	11 (34.4)	8 (23.5)	7 (35.0)	34 (29.8)	0.162
Depression	11 (39.3)	9 (28.1)	8 (23.5)	3 (15.0)	31 (27.2)	
Bipolar disorder	4 (14.3)	3 (9.4)	7 (20.6)	0 (0.0)	14 (12.3)	
Personality disorder	5 (17.9)	6 (18.8)	5 (14.7)	6 (30.0)	22 (19.3)	
Other	0 (0.0)	3 (9.4)	6 (17.6)	4 (20.0)	13 (11.4)	
Hospitalization type						
TSV	26 (92.9)	30 (93.8)	28 (82.4)	20 (100.0)	104 (91.2)	0.164
TSO	2 (7.1)	2 (6.3)	6 (17.6)	0 (0.0)	10 (8.8)	
Hospitalization days						
2–10	2 (7.1)	26 (81.3)	13 (38.2)	11 (61.1)	52 (46.4)	<0.001
11–20	13 (46.4)	5 (15.6)	13 (38.2)	2 (11.1)	33 (29.5)	
21–30	10 (35.7)	0 (0.0)	3 (8.8)	1 (5.6)	14 (12.5)	
31–40	2 (7.1)	0 (0.0)	2 (5.9)	0 (0.0)	4 (3.6)	
40+	1 (3.6)	1 (3.1)	3 (8.8)	4 (22.2)	9 (8.0)	
Previous hospitalization						
Yes	27 (96.4)	26 (81.3)	27 (79.4)	13 (65.0)	93 (81.6)	0.037
No	1 (3.6)	6 (18.8)	7 (20.6)	7 (35.0)	21 (18.4)	
Room type						
Single	1 (3.6)	2 (6.3)	4 (11.8)	5 (25.0)	12 (10.5)	0.108
Double	27 (96.4)	30 (93.8)	30 (88.2)	15 (75.0)	102 (89.5)	
Allowed to go out						
Yes	11 (39.3)	28 (87.5)	32 (94.1)	9 (45.0)	80 (70.2)	<0.001
No	17 (60.7)	4 (12.5)	2 (5.9)	11 (55.0)	34 (29.8)	
Smoke						
Yes	12 (42.9)	20 (62.5)	17 (50.0)	13 (65.0)	62 (54.4)	0.336
No	16 (57.1)	12 (37.5)	17 (50.0)	7 (35.0)	52 (45.6)	
Restraint						
Yes	0 (0.0)	2 (6.3)	1 (2.9)	0 (0.0)	3 (2.6)	0.554
No	28 (100.0)	30 (93.8)	33 (97.1)	20 (100.0)	111 (97.4)	
Visits allow						
Yes	24 (85.7)	29 (90.6)	33 (97.1)	17 (85.0)	103 (90.4)	0.324
No	4 (14.3)	3 (9.4)	1 (2.9)	3 (15.0)	11 (9.6)	

Data are presented as absolute number (%) and *P*‐value obtained by Fisher’s exact test. TSO, Involuntary admission (Trattamento Sanitario Obbligatorio); TSV, Voluntary admission (Trattamento Sanitario Volontario).

**Table 2:** Descriptive statistics of the staff members by unit

Unit	A (N = 16) (%)	B (N = 18) (%)	C (N = 45) (%)	D (N = 30) (%)	Total (N = 109)	*P*‐value
Gender						
Male	6 (42.9)	6 (40.0)	28 (62.2)	17 (56.7)	57 (54.8)	0.365
Female	8 (57.1)	9 (60.0)	17 (37.8)	13 (43.3)	47 (45.2)	
Age class						
21–30	1 (6.3)	0 (0.0)	2 (4.4)	5 (16.7)	8 (7.3)	0.460
31–40	0 (0.0)	2 (11.1)	6 (13.3)	2 (6.7)	10 (9.2)	
41–50	4 (25.0)	8 (44.4)	16 (35.6)	7 (23.3)	35 (32.1)	
51–60	9 (56.3)	6 (33.3)	15 (33.3)	12 (40.0)	42 (38.5)	
61–70	2 (12.5)	2 (11.1)	6 (13.3)	4 (13.3)	14 (12.8)	
Professional qualification						
Psychiatrist	3 (18.8)	10 (55.6)	12 (26.7)	6 (20.0)	31 (28.4)	<0.001
Psychologist	0 (0.0)	6 (33.3)	1 (2.2)	1 (3.3)	8 (7.3)	
Nurse	8 (50.0)	2 (11.1)	21 (46.7)	17 (56.7)	48 (44.0)	
Rehab therapist	0 (0.0)	0 (0.0)	1 (2.2)	2 (6.7)	3 (2.8)	
Nursing Assistant	5 (31.3)	0 (0.0)	7 (15.6)	3 (10.0)	15 (13.8)	
Other	0 (0.0)	0 (0.0)	3 (6.7)	1 (3.3)	4 (3.7)	
Education						
Primary/secondary	5 (31.3)	8 (44.4)	9 (20.0)	3 (10.0)	25 (22.9)	<0.001
BSc/nurse qualif	6 (37.5)	7 (38.9)	19 (42.2)	19 (63.3)	51 (46.8)	
MSc	2 (12.5)	0 (0.0)	0 (0.0)	8 (26.7)	10 (9.2)	
Specialization/other	3 (18.8)	3 (16.7)	17 (37.8)	0 (0.0)	23 (21.1)	
Years in MH						
<1	0 (0.0)	6 (33.3)	2 (4.4)	2 (7.1)	10 (9.3)	0.001
1–10	11 (68.8)	12 (66.7)	19 (42.2)	11 (39.3)	53 (49.5)	
11–20	1 (6.3)	0 (0.0)	12 (26.7)	5 (17.9)	18 (16.8)	
21–30	1 (6.3)	0 (0.0)	7 (15.6)	3 (10.7)	11 (10.3)	
31–40	3 (18.8)	0 (0.0)	5 (11.1)	7 (25.0)	15 (14.0)	
Years in MH						
Mean (SD)	12.5 (12.1)	1.4 (1.3)	14.3 (10.6)	15.0 (13.6)	12.1 (11.8)	<0.001
Years in APU						
<1	0 (0.0)	5 (27.8)	4 (8.9)	9 (32.1)	18 (16.8)	0.019
1–10	14 (87.5)	13 (72.2)	25 (55.6)	12 (42.9)	64 (59.8)	
11–20	0 (0.0)	0 (0.0)	10 (22.2)	4 (14.3)	14 (13.1)	
21–30	2 (12.5)	0 (0.0)	5 (11.1)	2 (7.1)	9 (8.4)	
31–40	0 (0.0)	0 (0.0)	1 (2.2)	1 (3.6)	2 (1.9)	
Years in APU						
Mean (SD)	8.5 (8.2)	1.2 (1.1)	9.5 (8.9)	6.6 (9.6)	7.2 (8.6)	<0.001

Data are presented as mean (SD: standard deviation) for quantitative variables and as absolute number (%) for categorical variables. *P*‐value obtained by Kruskal–Wallis test when comparing medians of quantitative variables, while Fisher’s exact test when comparing categorical ones. APU, Acute Psychiatric Unit; MH, Mental Health.

Two‐hundred and three EssenCES questionnaires were evaluated, with an overall response rate of 79% (74% for the patients and 85% for the staff members).

A total of 114 patients (from 20 to 34 in each unit) completed the EssenCES questionnaire. The majority of the participants were between 41 and 60 years old; 66 were male (58%); there was a larger proportion of males in all four units. Psychosis (30%) and depression (27%) were the most prevalent diagnoses; 91% were voluntary patients, and 82% have a previous history of hospitalization (Table 1).

One hundred and nine members of the staff employed at the four units completed the questionnaires. Most were nurses (44%), and many were psychiatrists (28%); there were also nursing assistants (14%), psychologists (7%) and rehab therapists (3%). The staff in one unit (B) had less years of experience in mental health or acute psychiatric units with respect to the other centres (Table 2).

### Social climate

The mean (SD) values for the EssenCES subscales and total score are outlined in Table 3. The scores of the patients and staff of each unit are outlined separately and all together. A significant difference between the centres was found in the scores for the *Experienced safety* subscale for both the patient and staff groups (*P* = 0.023 and *P* = 0.002 respectively). When the data of the patients and the staff were combined, the difference was even larger (*P* < 0.001) and the difference in the total EssenCES score between the units became significant (*P* = 0.009). Post hoc comparisons confirmed that unit D has significantly lower scores with respect to the other units.

**Table 3:** Summary statistics for the three subscales and total score for the EssenCES questionnaire by unit for patient, staff and the total sample

Unit	A	B	C	D	Total	*P*‐value
Patients	(N = 28)	(N = 32)	(N = 34)	(N = 20)	(N = 114)	
Patient cohesion	10.0 (3.3)	10.5 (3.5)	9.8 (3.5)	9.9 (4.4)	10.1 (3.6)	0.858
Experienced safety	14.1 (4.6)	15.1 (3.6)	12.9 (4.7)	11.1 (4.9)	13.5 (4.6)	0.023
Therapeutic hold	14.1 (3.7)	14.0 (3.0)	13.6 (3.5)	12.1 (5.5)	13.6 (3.9)	0.722
Total EssenCES score	38.3 (8.0)	39.7 (6.4)	36.3 (7.4)	33.0 (11.3)	37.2 (8.3)	0.084
Staff	(N = 16)	(N = 18)	(N = 45)	(N = 30)	(N = 109)	
Patient cohesion	9.9 (2.3)	8.4 (3.2)	9.6 (2.5)	9.8 (1.5)	9.5 (2.4)	0.328
Experienced safety	8.2 (2.4)	8.5 (3.1)	8.7 (3.2)	5.7 (3.5)	7.8 (3.4)	0.002
Therapeutic hold	14.9 (2.9)	14.2 (2.9)	14.6 (2.2)	16.1 (2.7)	15.0 (2.6)	0.054
Total EssenCES score	33.0 (4.6)	31.1 (4.4)	32.9 (4.4)	31.5 (4.2)	32.3 (4.4)	0.367
Total sample	(N = 44)	(N = 50)	(N = 79)	(N = 50)	(N = 223)	
Patient cohesion	10.0 (3.0)	9.8 (3.5)	9.7 (2.9)	9.9 (3.0)	9.8 (3.1)	0.893
Experienced safety	12.0 (4.9)	12.7 (4.7)	10.5 (4.4)	7.8 (4.9)	10.7 (5.0)	<0.001
Therapeutic hold	14.4 (3.4)	14.1 (3.0)	14.2 (2.9)	14.5 (4.5)	14.3 (3.4)	0.417
Total EssenCES score	36.3 (7.4)	36.6 (7.1)	34.4 (6.1)	32.1 (7.8)	34.8 (7.1)	0.009

Data are presented as mean (SD: standard deviation) and *P*‐value obtained by Kruskal–Wallis test.

**Fig. 1:**
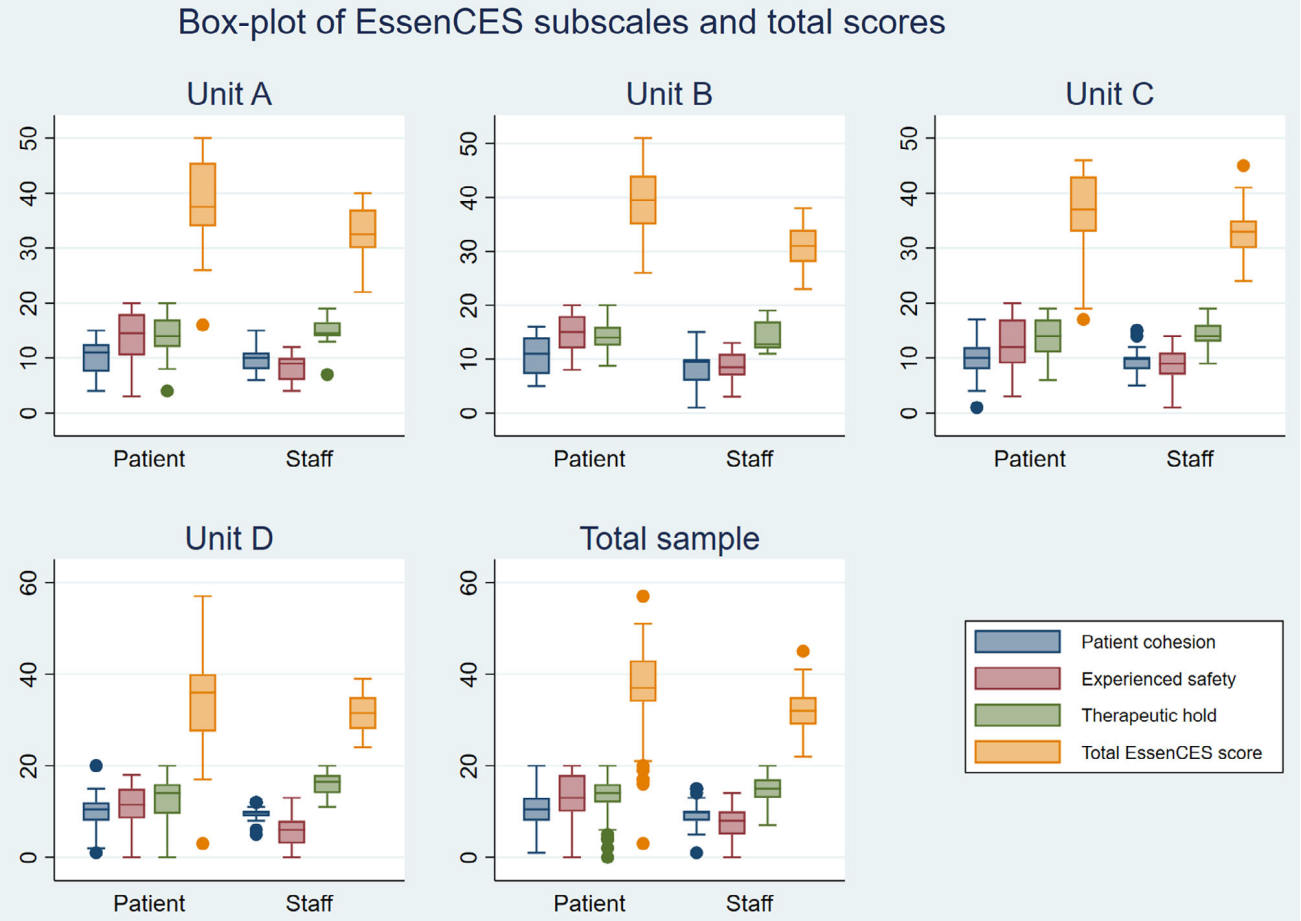
Box plots of the three subscales and total EssenCES score for patients and staff, separately for each unit and for the total sample. Note. In blue Patient cohesion; in red Experienced safety, in green Therapeutic hold and in orange the Total EssenCES score.

Figure 1 presents box plots of the three subscales and the total EssenCES score of the patient and staff groups for each unit and for the total sample. In Tables S1a–S1d, the absolute numbers and percentages of each item in each of the four units are shown. The patients had significantly higher scores than the staff for the *Experienced safety* subscale at all four units (Cohen’s *d*: A = 1.50, B = 1.91, C = 1.06, D = 1.31; all *P* < 0.001). Moreover in all but one unit (D), patients had significantly higher values than staff in the total EssenCES score (A: *d* = 0.75, *P* = 0.016; B: *d* = 1.48, *P* < 0.001; C: *d* = 0.57, *P* = 0.003). Conversely, in one unit (D), the staff had significantly higher scores than the patients for the *Therapeutic hold* subscale (*d* = 0.99, *P* = 0.005). There was also a marginally significant difference in one unit (B) for the *Patient cohesion* subscale (*d* = 0.63, *P* = 0.055).

In one unit (B), the patients assigned to single rooms had higher scores on the *Experienced safety* subscale than those in double rooms (*d* = 1.51, *P* = 0.034) (data not shown).

The patients in the units where many activities were organized had significantly lower scores in *Experienced safety* and the total EssenCES score (Table 4) (*d *= −0.55, *P* = 0.006 and *d *= −0.48, *P* = 0.025 respectively). More hours available for visits resulted in a significantly higher score for the *Experienced safety* subscale (*d* = 0.49, *P* = 0.024) and marginally higher score for total EssenCES score (*d* = 0.43, *P* = 0.070). Those patients residing in rural areas had higher values in *the Experienced safety* and in the total EssenCES scores with respect to those living in urban areas (*d* = 0.55, *P* = 0.006 and *d* = 0.48, *P* = 0.025 respectively); likewise, those who could use the cell phone for 12–13 h per day had higher scores with respect to those who could use them only a few hours a day (*P* = 0.012 and *P* = 0.043 respectively).

**Table 4:** Summary statistics for the three subscales and total EssenCES score by contextual characteristics for patients and staff

	Patient cohesion	Experienced safety	Therapeutic hold	Total EssenCES score
Patients				
Garden				
Yes (N = 48)	10.0 (3.8)	12.9 (5.0)	13.3 (4.6)	36.1 (9.7)
No (N = 66)	10.2 (3.5)	14.0 (4.3)	13.8 (3.3)	37.9 (7.1)
Activities		*P* = 0.006*		*P* = 0.025*
Yes (N = 54)	9.9 (3.8)	12.2 (4.8)	13.0 (4.4)	35.1 (9.1)
No (N = 60)	10.3 (3.4)	14.7 (4.1)	14.1 (3.3)	39.0 (7.2)
Visits hours		*P* = 0.024*		*P* = 0.070*
2 h/die (N = 82)	9.9 (3.6)	12.9 (4.8)	13.4 (4.2)	36.2 (8.8)
10 h/die (N = 32)	10.5 (3.5)	15.1 (3.6)	14.0 (3.0)	39.7 (6.4)
Cell phone use		*P* = 0.011**		*P* = 0.043**
2 h/die (N = 34)	9.8 (3.5)	12.9 (4.7)	13.6 (3.5)	36.3 (7.4)
12–13 h/die (N = 60)	10.3 (3.4)	14.7 (4.1)	14.1 (3.3)	39.0 (7.2)
24 h/die (N = 20)	9.9 (4.4)	11.1 (4.9)	12.1 (5.5)	33.0 (11.3)
Location		*P* = 0.006*		*P* = 0.025*
Rural (N = 60)	10.3 (3.4)	14.7 (4.1)	14.1 (3.3)	39.0 (7.2)
Urban (N = 54)	9.9 (3.8)	12.2 (4.8)	13.0 (4.4)	35.1 (9.1)
Staff				
Garden		*P* = 0.002*	*P* = 0.015*	
Yes (N = 46)	9.8 (1.8)	6.5 (3.3)	15.7 (2.8)	32.0 (4.3)
No (N = 63)	9.2 (2.7)	8.7 (3.2)	14.5 (2.4)	32.4 (4.4)
Activities				
Yes (N = 75)	9.7 (2.1)	7.5 (3.6)	15.2 (2.5)	32.4 (4.3)
No (N = 34)	9.1 (2.9)	8.4 (2.7)	14.5 (2.9)	32.0 (4.6)
Visits hours	*P* = 0.090*		*P* = 0.099*	
2 h/die (N = 91)	9.7 (2.1)	7.6 (3.4)	15.2 (2.5)	32.5 (4.4)
10 h/die (N = 18)	8.4 (3.2)	8.5 (3.1)	14.2 (2.9)	31.1 (4.4)
Cell phone use		*P* = 0.001**	*P* = 0.042**	
2 h/die (N = 45)	9.6 (2.5)	8.7 (3.2)	14.6 (2.2)	32.9 (4.4)
12–13 h/die (N = 34)	9.1 (2.9)	8.4 (2.7)	14.5 (2.9)	32.0 (4.6)
24 h/die (N = 30)	9.8 (1.5)	5.7 (3.5)	16.1 (2.7)	31.5 (4.2)
Location				
Rural (N = 34)	9.1 (2.9)	8.4 (2.7)	14.5 (2.9)	32.0 (4.6)
Urban (N = 75)	9.7 (2.1)	7.5 (3.6)	15.2 (2.5)	32.4 (4.3)

Data are presented as mean (SD: standard deviation) and *P*‐value obtained by *Mann–Whitney test or **Kruskal–Wallis test. Only *P*‐values < 0.10 are reported.

No significant differences in the scores were found with regard to the type of hospitalization, the presence of previous hospitalizations, smoking behaviours or use of restraint.

Regarding the staff members, working in a unit with a garden resulted in significantly lower scores for the *Experienced safety* subscale (*d *= −0.66, *P* = 0.002), but significantly higher ones for the *Therapeutic hold* subscale (*d* = 0.46, *P* = 0.015) (Table 4). The rule permitting patients to spend more time on the phone resulted in a significantly lower score on the *Experienced safety* subscale (24 h/day vs 2 h/day *d *= −0.92 and 24 h/day vs 12–13 h/day *d *= −0.87, *P* = 0.001) and in a significantly higher score for *Therapeutic hold* subscale (24 h/day vs 2 h/day *d* = 0.60 and 24 h/day vs 12–13 h/day *d* = 0.54, *P* = 0.042) for the staff members.

The *Therapeutic hold* subscale for the sample as a whole was weakly correlated with the *Patient cohesion* subscale (*r* = 0.25, *P* < 0.001). The correlation was higher in the patient group with respect to that in the staff group (*r* = 0.35, *P* < 0.001; *r* = 0.22, *P* = 0.0194 respectively). No significant correlation was found between the *Experienced safety* and the *Patient cohesion* subscales. A weak negative correlation was found between the *Therapeutic hold* and the *Experienced safety* subscales in the whole sample (*r* = −0.20, *P* = 0.0029). The effect was mainly attributed to the staff sample (*r* = −0.39, *P* < 0.001); the correlation was not significant in the patient sample.

### Factor analysis and internal consistency

Factor analysis followed by a varimax rotation (Kaiser–Meyer–Olkin Measure of sampling adequacy = 0.802, Bartlett’s test for sphericity *P* < 0.001) revealed three eigenvalues above 1 as in the original structure. In the predicted three‐factor solution, item loadings ranged between 0.62 and 0.82 for factor 1 (*Experienced safety*), between 0.58 and 0.69 for factor 2 (*Patient cohesion*) and between 0.38 and 0.74 for factor 3 (*Therapeutic hold*) (Table S2).

Table 5 shows the internal consistency of the three subscales of the EssenCES and the total score for the whole sample. Cronbach's α ranged between 0.77 and 0.79 for the patients, between 0.72 and 0.83 for the staff and between 0.75 and 0.87 for the whole sample. Since all Cronbach's alpha values exceeded 0.70, this would indicate that the Italian translation of the EssenCES has a satisfactory internal consistency. Moreover, the CITC values for the EssenCES subscales for the combined sample ranged between 0.38 and 0.70 (mean = 0.60), while the CITC values for the total EssenCES scale ranged between 0.11 and 0.57 (mean = 0.35).

**Table 5:** Internal consistency (Cronbach’s α) of EssenCES subscale and total scale for patients, staff and total sample

Scale	Patients (N = 114)	Staff (N = 109)	Total (N = 223)
Patient cohesion	0.77	0.83	0.79
Experienced safety	0.79	0.83	0.87
Therapeutic hold	0.77	0.72	0.76
Total EssenCES score	0.78	0.79	0.75

## Discussion

One of the study’s salient findings, which was found in all four units, was the difference in the perception of safety between the patients and the staff members. Moreover, with one exception, the patients perceived a better climate than the staff did. The differences in the perception of the staff and the patients were not surprising as other studies have reported similar findings (BootsMiller *et al*. 1997; Efkemann *et al*. 2019; Hottinen *et al*. 2020; Schalast & Sieß, 2018). In fact, the discrepancy between nurse and patient perceptions is not unique to psychiatric settings as even nurses working in other fields of medicine and other types of wards have remarked about it (Papastavrou *et al*. 2011).

From a general perspective, safety was the only subscale that led to significant differences in the scores linked to the contextual variables of our study which were activities such as being able to: receive visitors, use mobile phones, spend time away from the ward and participate in different activities. Stricter or more relaxed rules regarding these activities affected the scores of both the staff members and the patients. Safety during their stay as inpatients seemed to be an important concern of the patients studied. Although these preoccupations have rarely if at all been reported by other studies, it is in any case a very important consideration that can affect that therapeutic outcomes (McAndrew *et al*. 2014; Muir‐Cochrane & Gerace, 2015; Pelto‑Piri *et al*. 2019).

Another important finding was that the patients who were hospitalized in wards where they were allowed to have more visiting hours and more time to use the mobile phone had higher scores on the *Experienced Safety* subscale. But the staff members, and in particular the nurses, had a completely different perspective: while the patients with the possibility of spending time away from the ward and of being able to use their mobile phone several hours a day have scores that indicate a better perception of safety, these same variables lead to opposite scores in the staff showing a perception of less safety.

It should be pointed out that these apparently irrelevant aspects of routine ‘hospital life’ are rarely mentioned in studies published in the literature. Instead, there is growing interest in the potential of using mobile phones to support the treatment of psychiatric disorders, such as schizophrenia (Firth *et al*. 2016) or depression, or in the role of the smartphone in healthcare.

The only study focussing on the outcome of allowing psychiatric inpatients to use smartphones was carried out by O’Connor *et al*. (2018) who evaluated the inpatients of some New South Wales psychiatric units whose access to smartphones or other electronic devices was denied in 85% of the units. In the study of O’Connor *et al*. (2018), there was a wide gap in the perception of the patients and staff members: 75% of the patients thought that they should be able to access their mobile phones, but only 30% of the staff thought it was a good idea to let the patients use them. While the nurses’ major concern was fear of being photographed or recorded, the patients were interested in maintaining their social connections. In our study, three units denied the free use of smartphones and each unit had established different rules, but as we stated previously the patients of those units with less strict rules on mobile phones have a better feeling of safety.

In their review, Papadopoulos *et al*. (2012) pointed out that denied requests that at times led to aggression and violence referred to phone calls, but the meta‐analysis found only one article that made mention to this problem. As opposed to the past, patients are no longer seeking to use the phone (Brière *et al*. 2012; O’Connor *et al*. 2018) but are asking to use their smartphone, a complex, sophisticated, ubiquitous instrument. Making reference to teenage inpatients, Brière *et al*. (2012) pointed out that they preferred losing their wallet (identity card and credit card) than their smartphone. The comment recognizes the strong link that exists in many between the smartphone and one’s identity, memory and self‐image, all therapeutic issues in psychiatric patients (Brière *et al*. 2012; Jenkins *et al*. 2021).

As Kalagi *et al*. (2018) demonstrated, being able to leave the unit contributed to giving the patients a better perception of the ward climate. In their review investigating aggression in psychiatric settings, Papadopoulos, *et al*. (2012) found that limiting the patients freedom by placing restrictions on leaving the ward was the most frequent cause of aggression. In fact there appears to be a clear relationship between locked door policies, strict rules and aggression (Alexander & Bowers, 2004; Bowers *et al*. 2010; Efkemann *et al*. 2019; Papadopoulos *et al*. 2012; Schalast & Sieß, 2018), but in our study patients who could go out had lower scores on total EssenCES than those who could not. Maybe the low mean length of stay, the 46.4% of the patients fall within the range of 2–10 days of hospitalization, can have contributed to this finding.

As far as the garden was concerned, while psychiatric settings having the possibility of spending time in a garden seemed to play a key role in mental health contributing to better outcomes (Connellan *et al*. 2013; Kalagi *et al*. 2018), their presence in two units (A and D) did not seem to produce a significant difference in the perception of the ward climate. It is also true that the staff members of those two units did have a higher sense of *Therapeutic hold*, although they had a lower perception of safety.

It is interesting, in the light of what has been said above, that a negative correlation was found between *Therapeutic hold* and *Experienced safety* subscales, mainly due to the staff’s negative perception. Although the correlation is a weak one, it is nevertheless statistically significant and worthy of discussion given its implications. According to the nurses, when they carry out activities they consider therapeutic, their perceived safety decreases, and vice versa. This finding highlights the nurses’ concern about how some activities and rules can affect safety and how they themselves may be placed in a position of having to manage the risk of aggression and violence. These are findings similar to those described in the ethnographic study of McKeown *et al*. (2019) and to the nursing management of ‘permeability’ as shown by Quirk *et al*. (2006).

Nurses often complain about excessively flexible rules (i.e. unlocked door policies or permitting patients to use smartphones) because they find that lax rules create a more difficult, less safe situation that they have to manage forcing them to take on duties that are not part of their nursing competencies (Kalagi *et al*. 2018). Admitting visitors, locking/unlocking doors, taking patients for walks in the garden and checking how patients are using their smartphones are added to nurses’ other duties (McAndrew *et al*. 2014) and activities linked to risk management (Clancy & Happell, 2014; McKeown *et al*. 2019).

Our study shows that the nurses seem primarily concerned with how some activities and rules can affect safety and how they themselves may be placed in a position of having to manage the risk of aggression and violence, indeed in the paragraph above it is described the negative discrepancy between *Therapeutic hold* and *Experienced safety* expressed by nurses, with regard to the ward’s rules and routine.

This study also highlights the importance that patients attach to the use of smartphones and how this is not just a way for patients to spend time (important thing anyway) but it affects their feeling of security, self‐perception, central elements in inpatient experience. To the best of our knowledge, this is the first study that, despite its limitations, brings out this correlation clearly.

The study has some limitations that should be highlighted. The first is that it was carried out in four psychiatric units, all located in the same geographic area and thus tied to a specific cultural and clinical milieu and not representative of the entire Italian population. Another was the small sample sizes of the two populations (patients and staff members) which prevents us from making generalizations about the data. Although the EssenCES‐IT has been validated linguistically, but not statistically, its application in acute non forensic psychiatric settings has not been thoroughly studied. It is nevertheless true that the internal consistency for the Italian translation of the EssenCES was found to be satisfactory in this study.

## Conclusions

Visiting hours, smartphones, gardens and activities are all occasions for the patients to interact with one another, with external visitors (McAllister *et al*. 2021) and with the staff, and all of these possibilities can contribute to the ward’s climate and therapeutic relationships.

It is important that healthcare administrators bridge the gap between how the staff and the patients perceive the unit’s climate because it could lead to strategies and decisions that could promote the ward’s climate and the quality of care. If nurses feel unsafe, this could lead, just as it has in the past, to stricter rules which could trigger a vicious circle of violence and aggression. While there are numerous studies that have investigated the patient’s point of view, the therapeutic implications regarding ward’s rules seem to have been brushed aside by the fear of violence and aggression.

The ‘anxious vigilance’ described by Muir‐Cochrane *et al*. (2012) is closely linked to the concept of the ‘anxious profession’ described by Lakerman (2006) as well as to the ‘culture of fear and watchfulness’ reported by Clancy and Happell (2014) and, finally, to Jones’ (2020) comment that ‘risk assessment becomes the crux of mental health practice rather than an outcome of good quality care’.

It is important that we once again consider the patient who exhibits aggressive behaviours as someone who is going through a crisis (from the ancient Greek word *krinein*‐divide, separate but also changing) and not just a person whose aggression needs to be managed (Clancy & Happell, 2014). An approach such as this will lead us to consider the management of aggression as an opportunity to create a therapeutic relationship within the context of the unit’s rules and routine and to enhance the ward’s climate using a patient‐centred care approach.

Lastly, although the EssenCES‐IT needs Italian statistical validation, it seems to be a promising tool for evaluating the social climate of small acute units with short‐medium stays as are most Italian ones.

## Relevance for Clinical Practice

By this multicentric observational cohort study, we found a gap between the patients and staff members’ perceptions of safety which seemed to be affected by the rules of the ward, in particular the number of visiting hours and possibility of having a smartphone.

Strict rules, especially when the patients consider them unfair, may have a negative effect triggering a vicious circle leading to more aggression. The rules of a psychiatric ward, which are an important aspect of ward climate, need to be made not only in consideration of risk management but also of a patient‐centred care approach.

## Funding

The authors received no financial support for the research, authorship and/or publication of this article.

## Ethics approval

Formal approval of the study was obtained from the Italian Health Authorities and the Local Departments of Mental Health.

## Supporting information

Table S1a Summary statistics, unit A
Table S1b Summary statistics, unit B
Table S1c Summary statistics, unit C
Table S1d Summary statistics, unit D
Table S2 Factor loadings following principal components analysis using orthogonal varimax rotation
