# Supplementary material for: Rules and ward climate in acute psychiatric setting: Comparison of staff and patient perceptions
Source: Int J Ment Health Nurs. 2022 Feb 6;31(3):611–24. doi: 10.1111/inm.12980 (PMC9305954; doi:10.1111/inm.12980)
Supplement: Supplementary file 1 — Table S1a Summary statistics, unit A Table S1b Summary statistics, unit B Table S1c Summary statistics, unit C Table S1d Summary statistics, unit D Table S2 Factor loadings following principal components analysis using orthogonal varimax rotation [file INM-31-611-s001.docx]

**Supplementary Table 1a: Summary statistics, unit A**

| \|  \| Patient \| Staff \|  \| \| --- \| --- \| --- \| --- \| \|  \| (N = 28) \| (N = 16) \| p-value \| |
| --- | --- | --- | --- | --- | --- | --- | --- | --- |
| \| **CP1_2** \|  \|  \| 0.416 \| \| --- \| --- \| --- \| --- \| \| little \| 8 (28.6%) \| 2 (12.5%) \|  \| \| somewhat \| 11 (39.3%) \| 10 (62.5%) \|  \| \| quite a lot \| 7 (25.0%) \| 4 (25.0%) \|  \| \| very much \| 2 (7.1%) \| 0 (0.0%) \|  \| \| **CP2_5** \|  \|  \| 0.076 \| \| not at all \| 5 (17.9%) \| 0 (0.0%) \|  \| \| little \| 4 (14.3%) \| 1 (6.3%) \|  \| \| somewhat \| 10 (35.7%) \| 12 (75.0%) \|  \| \| quite a lot \| 9 (32.1%) \| 3 (18.8%) \|  \| \| **CP3_8** \|  \|  \| 0.127 \| \| not at all \| 1 (3.6%) \| 0 (0.0%) \|  \| \| little \| 4 (14.3%) \| 4 (25.0%) \|  \| \| somewhat \| 10 (35.7%) \| 10 (62.5%) \|  \| \| quite a lot \| 12 (42.9%) \| 2 (12.5%) \|  \| \| very much \| 1 (3.6%) \| 0 (0.0%) \|  \| \| **CP4_11** \|  \|  \| 0.176 \| \| not at all \| 6 (21.4%) \| 0 (0.0%) \|  \| \| little \| 6 (21.4%) \| 5 (31.3%) \|  \| \| somewhat \| 11 (39.3%) \| 10 (62.5%) \|  \| \| quite a lot \| 4 (14.3%) \| 1 (6.3%) \|  \| \| very much \| 1 (3.6%) \| 0 (0.0%) \|  \| \| **CP5_14** \|  \|  \| 0.127 \| \| little \| 7 (25.0%) \| 3 (18.8%) \|  \| \| somewhat \| 10 (35.7%) \| 11 (68.8%) \|  \| \| quite a lot \| 9 (32.1%) \| 1 (6.3%) \|  \| \| very much \| 2 (7.1%) \| 1 (6.3%) \|  \| \| **Patient cohesion** \|  \|  \| 0.617 \| \| Median (Q1, Q3) \| 11.0 (7.5, 12.5) \| 10.0 (8.0, 11.0) \|  \| \| **SP1_3** \|  \|  \| <0.001 \| \| very much \| 2 (7.1%) \| 2 (12.5%) \|  \| \| quite a lot \| 2 (7.1%) \| 6 (37.5%) \|  \| \| somewhat \| 3 (10.7%) \| 8 (50.0%) \|  \| \| little \| 5 (17.9%) \| 0 (0.0%) \|  \| \| not at all \| 16 (57.1%) \| 0 (0.0%) \|  \| \| **SP2_6** \|  \|  \| <0.001 \| \| very much \| 3 (10.7%) \| 1 (6.3%) \|  \| \| quite a lot \| 2 (7.1%) \| 3 (18.8%) \|  \| \| somewhat \| 2 (7.1%) \| 11 (68.8%) \|  \| \| little \| 6 (21.4%) \| 1 (6.3%) \|  \| \| not at all \| 15 (53.6%) \| 0 (0.0%) \|  \| \| **SP3_9** \|  \|  \| <0.001 \| \| very much \| 1 (3.6%) \| 2 (12.5%) \|  \| \| quite a lot \| 2 (7.1%) \| 1 (6.3%) \|  \| \| somewhat \| 7 (25.0%) \| 13 (81.3%) \|  \| \| little \| 7 (25.0%) \| 0 (0.0%) \|  \| \| not at all \| 11 (39.3%) \| 0 (0.0%) \|  \| \| **SP4_12** \|  \|  \| 0.001 \| \| very much \| 1 (3.6%) \| 1 (6.3%) \|  \| \| quite a lot \| 1 (3.6%) \| 2 (12.5%) \|  \| \| somewhat \| 9 (32.1%) \| 10 (62.5%) \|  \| \| little \| 2 (7.1%) \| 3 (18.8%) \|  \| \| not at all \| 15 (53.6%) \| 0 (0.0%) \|  \| \| **SP5_15** \|  \|  \| 0.051 \| \| very much \| 5 (17.9%) \| 2 (12.5%) \|  \| \| quite a lot \| 5 (17.9%) \| 5 (31.3%) \|  \| \| somewhat \| 8 (28.6%) \| 9 (56.3%) \|  \| \| little \| 2 (7.1%) \| 0 (0.0%) \|  \| \| not at all \| 8 (28.6%) \| 0 (0.0%) \|  \| \| **Experienced safety** \|  \|  \| <0.001 \| \| Median (Q1, Q3) \| 14.5 (10.5, 18.0) \| 9.0 (6.0, 10.0) \|  \| \| **ST1_4** \|  \|  \| 0.166 \| \| not at all \| 2 (7.1%) \| 0 (0.0%) \|  \| \| somewhat \| 7 (25.0%) \| 1 (6.3%) \|  \| \| quite a lot \| 9 (32.1%) \| 10 (62.5%) \|  \| \| very much \| 10 (35.7%) \| 5 (31.3%) \|  \| \| **ST2_7** \|  \|  \| 0.962 \| \| not at all \| 2 (7.1%) \| 1 (6.3%) \|  \| \| little \| 1 (3.6%) \| 1 (6.3%) \|  \| \| somewhat \| 5 (17.9%) \| 4 (25.0%) \|  \| \| quite a lot \| 13 (46.4%) \| 7 (43.8%) \|  \| \| very much \| 7 (25.0%) \| 3 (18.8%) \|  \| \| **ST3_10** \|  \|  \| 0.702 \| \| not at all \| 2 (7.1%) \| 0 (0.0%) \|  \| \| somewhat \| 7 (25.0%) \| 3 (18.8%) \|  \| \| quite a lot \| 16 (57.1%) \| 10 (62.5%) \|  \| \| very much \| 3 (10.7%) \| 3 (18.8%) \|  \| \| **ST4_13** \|  \|  \| 0.907 \| \| very much \| 1 (3.6%) \| 0 (0.0%) \|  \| \| quite a lot \| 2 (7.1%) \| 1 (6.3%) \|  \| \| somewhat \| 5 (17.9%) \| 4 (25.0%) \|  \| \| little \| 7 (25.0%) \| 2 (12.5%) \|  \| \| not at all \| 13 (46.4%) \| 9 (56.3%) \|  \| \| **ST5_16** \|  \|  \| 0.659 \| \| not at all \| 1 (3.6%) \| 0 (0.0%) \|  \| \| little \| 1 (3.6%) \| 1 (6.3%) \|  \| \| somewhat \| 9 (32.1%) \| 3 (18.8%) \|  \| \| quite a lot \| 10 (35.7%) \| 9 (56.3%) \|  \| \| very much \| 7 (25.0%) \| 3 (18.8%) \|  \| \| **Therapeutic hold** \|  \|  \| 0.421 \| \| Median (Q1, Q3) \| 14.0 (12.0, 17.0) \| 14.5 (14.0, 16.5) \|  \| \| **Total EssenCES score** \|  \|  \| 0.016 \| \| Median (Q1, Q3) \| 37.5 (34.0, 45.5) \| 32.5 (30.0, 37.0) \|  \| |

**Supplementary Table 1b: Summary statistics, unit B**

| \|  \| Patient \| Staff \|  \| \| --- \| --- \| --- \| --- \| \|  \| (N = 32) \| (N = 18) \| p-value \| |
| --- | --- | --- | --- | --- | --- | --- | --- | --- |
| \| **CP1_2** \|  \|  \| 0.224 \| \| --- \| --- \| --- \| --- \| \| not at all \| 0 (0.0%) \| 1 (5.6%) \|  \| \| little \| 6 (18.8%) \| 7 (38.9%) \|  \| \| somewhat \| 17 (53.1%) \| 7 (38.9%) \|  \| \| quite a lot \| 6 (18.8%) \| 3 (16.7%) \|  \| \| very much \| 3 (9.4%) \| 0 (0.0%) \|  \| \| **CP2_5** \|  \|  \| 0.019 \| \| little \| 8 (25.0%) \| 6 (33.3%) \|  \| \| somewhat \| 9 (28.1%) \| 11 (61.1%) \|  \| \| quite a lot \| 11 (34.4%) \| 1 (5.6%) \|  \| \| very much \| 4 (12.5%) \| 0 (0.0%) \|  \| \| **CP3_8** \|  \|  \| 0.143 \| \| not at all \| 4 (12.5%) \| 1 (5.6%) \|  \| \| little \| 10 (31.3%) \| 6 (33.3%) \|  \| \| somewhat \| 8 (25.0%) \| 10 (55.6%) \|  \| \| quite a lot \| 8 (25.0%) \| 1 (5.6%) \|  \| \| very much \| 2 (6.3%) \| 0 (0.0%) \|  \| \| **CP4_11** \|  \|  \| 0.210 \| \| not at all \| 4 (12.5%) \| 1 (5.6%) \|  \| \| little \| 9 (28.1%) \| 5 (27.8%) \|  \| \| somewhat \| 11 (34.4%) \| 11 (61.1%) \|  \| \| quite a lot \| 8 (25.0%) \| 1 (5.6%) \|  \| \| **CP5_14** \|  \|  \| 0.002 \| \| not at all \| 0 (0.0%) \| 1 (5.6%) \|  \| \| little \| 4 (12.9%) \| 4 (22.2%) \|  \| \| somewhat \| 9 (29.0%) \| 12 (66.7%) \|  \| \| quite a lot \| 16 (51.6%) \| 1 (5.6%) \|  \| \| very much \| 2 (6.5%) \| 0 (0.0%) \|  \| \| **Patient cohesion** \|  \|  \| 0.056 \| \| Median (Q1, Q3) \| 11.0 (7.3, 14.0) \| 9.5 (6.0, 10.0) \|  \| \| **SP1_3** \|  \|  \| 0.008 \| \| very much \| 2 (6.3%) \| 4 (22.2%) \|  \| \| quite a lot \| 3 (9.4%) \| 5 (27.8%) \|  \| \| somewhat \| 4 (12.5%) \| 5 (27.8%) \|  \| \| little \| 9 (28.1%) \| 3 (16.7%) \|  \| \| not at all \| 14 (43.8%) \| 1 (5.6%) \|  \| \| **SP2_6** \|  \|  \| <0.001 \| \| very much \| 0 (0.0%) \| 2 (11.1%) \|  \| \| quite a lot \| 3 (9.4%) \| 3 (16.7%) \|  \| \| somewhat \| 3 (9.4%) \| 8 (44.4%) \|  \| \| little \| 9 (28.1%) \| 5 (27.8%) \|  \| \| not at all \| 17 (53.1%) \| 0 (0.0%) \|  \| \| **SP3_9** \|  \|  \| <0.001 \| \| quite a lot \| 1 (3.1%) \| 6 (33.3%) \|  \| \| somewhat \| 7 (21.9%) \| 11 (61.1%) \|  \| \| little \| 9 (28.1%) \| 1 (5.6%) \|  \| \| not at all \| 15 (46.9%) \| 0 (0.0%) \|  \| \| **SP4_12** \|  \|  \| <0.001 \| \| quite a lot \| 3 (9.4%) \| 2 (11.1%) \|  \| \| somewhat \| 5 (15.6%) \| 13 (72.2%) \|  \| \| little \| 6 (18.8%) \| 3 (16.7%) \|  \| \| not at all \| 18 (56.3%) \| 0 (0.0%) \|  \| \| **SP5_15** \|  \|  \| 0.006 \| \| very much \| 1 (3.1%) \| 6 (33.3%) \|  \| \| quite a lot \| 6 (18.8%) \| 3 (16.7%) \|  \| \| somewhat \| 11 (34.4%) \| 7 (38.9%) \|  \| \| little \| 4 (12.5%) \| 2 (11.1%) \|  \| \| not at all \| 10 (31.3%) \| 0 (0.0%) \|  \| \| **Experienced safety** \|  \|  \| <0.001 \| \| Median (Q1, Q3) \| 15.0 (12.0, 18.0) \| 8.5 (7.0, 11.0) \|  \| \| **ST1_4** \|  \|  \| 0.772 \| \| not at all \| 2 (6.3%) \| 0 (0.0%) \|  \| \| little \| 1 (3.1%) \| 0 (0.0%) \|  \| \| somewhat \| 10 (31.3%) \| 4 (22.2%) \|  \| \| quite a lot \| 11 (34.4%) \| 9 (50.0%) \|  \| \| very much \| 8 (25.0%) \| 5 (27.8%) \|  \| \| **ST2_7** \|  \|  \| 0.195 \| \| little \| 1 (3.1%) \| 1 (5.6%) \|  \| \| somewhat \| 5 (15.6%) \| 6 (33.3%) \|  \| \| quite a lot \| 18 (56.3%) \| 10 (55.6%) \|  \| \| very much \| 8 (25.0%) \| 1 (5.6%) \|  \| \| **ST3_10** \|  \|  \| 0.762 \| \| not at all \| 1 (3.1%) \| 0 (0.0%) \|  \| \| little \| 4 (12.5%) \| 1 (5.6%) \|  \| \| somewhat \| 6 (18.8%) \| 3 (16.7%) \|  \| \| quite a lot \| 17 (53.1%) \| 9 (50.0%) \|  \| \| very much \| 4 (12.5%) \| 5 (27.8%) \|  \| \| **ST4_13** \|  \|  \| 0.322 \| \| very much \| 1 (3.1%) \| 0 (0.0%) \|  \| \| quite a lot \| 1 (3.1%) \| 1 (5.9%) \|  \| \| somewhat \| 9 (28.1%) \| 3 (17.6%) \|  \| \| little \| 4 (12.5%) \| 6 (35.3%) \|  \| \| not at all \| 17 (53.1%) \| 7 (41.2%) \|  \| \| **ST5_16** \|  \|  \| 0.131 \| \| not at all \| 1 (3.2%) \| 0 (0.0%) \|  \| \| little \| 3 (9.7%) \| 0 (0.0%) \|  \| \| somewhat \| 10 (32.3%) \| 11 (61.1%) \|  \| \| quite a lot \| 9 (29.0%) \| 6 (33.3%) \|  \| \| very much \| 8 (25.8%) \| 1 (5.6%) \|  \| \| **Therapeutic hold** \|  \|  \| 0.976 \| \| Median (Q1, Q3) \| 14.0 (12.5, 16.0) \| 12.8 (12.0, 17.0) \|  \| \| **Total EssenCES score** \|  \|  \| <0.001 \| \| Median (Q1, Q3) \| 39.5 (35.0, 44.0) \| 31.0 (28.0, 34.0) \|  \| |

**Supplementary Table 1c: Summary statistics, unit C**

| \|  \| Patient \| Staff \|  \| \| --- \| --- \| --- \| --- \| \|  \| (N = 34) \| (N = 45) \| p-value \| |
| --- | --- | --- | --- | --- | --- | --- | --- | --- |
| \| **CP1_2** \|  \|  \| 0.026 \| \| --- \| --- \| --- \| --- \| \| not at all \| 2 (5.9%) \| 0 (0.0%) \|  \| \| little \| 9 (26.5%) \| 8 (17.8%) \|  \| \| somewhat \| 13 (38.2%) \| 31 (68.9%) \|  \| \| quite a lot \| 9 (26.5%) \| 6 (13.3%) \|  \| \| very much \| 1 (2.9%) \| 0 (0.0%) \|  \| \| **CP2_5** \|  \|  \| 0.319 \| \| little \| 9 (26.5%) \| 12 (26.7%) \|  \| \| somewhat \| 13 (38.2%) \| 25 (55.6%) \|  \| \| quite a lot \| 10 (29.4%) \| 7 (15.6%) \|  \| \| very much \| 2 (5.9%) \| 1 (2.2%) \|  \| \| **CP3_8** \|  \|  \| 0.271 \| \| not at all \| 4 (11.8%) \| 1 (2.2%) \|  \| \| little \| 9 (26.5%) \| 11 (24.4%) \|  \| \| somewhat \| 13 (38.2%) \| 24 (53.3%) \|  \| \| quite a lot \| 7 (20.6%) \| 9 (20.0%) \|  \| \| very much \| 1 (2.9%) \| 0 (0.0%) \|  \| \| **CP4_11** \|  \|  \| 0.814 \| \| not at all \| 3 (8.8%) \| 2 (4.4%) \|  \| \| little \| 6 (17.6%) \| 9 (20.0%) \|  \| \| somewhat \| 19 (55.9%) \| 28 (62.2%) \|  \| \| quite a lot \| 5 (14.7%) \| 6 (13.3%) \|  \| \| very much \| 1 (2.9%) \| 0 (0.0%) \|  \| \| **CP5_14** \|  \|  \| 0.088 \| \| not at all \| 2 (5.9%) \| 0 (0.0%) \|  \| \| little \| 5 (14.7%) \| 10 (22.2%) \|  \| \| somewhat \| 16 (47.1%) \| 28 (62.2%) \|  \| \| quite a lot \| 9 (26.5%) \| 7 (15.6%) \|  \| \| very much \| 2 (5.9%) \| 0 (0.0%) \|  \| \| **Patient cohesion** \|  \|  \| 0.498 \| \| Median (Q1, Q3) \| 10.0 (8.0, 12.0) \| 10.0 (8.0, 10.0) \|  \| \| **SP1_3** \|  \|  \| <0.001 \| \| very much \| 2 (5.9%) \| 10 (22.2%) \|  \| \| quite a lot \| 3 (8.8%) \| 13 (28.9%) \|  \| \| somewhat \| 8 (23.5%) \| 18 (40.0%) \|  \| \| little \| 10 (29.4%) \| 4 (8.9%) \|  \| \| not at all \| 11 (32.4%) \| 0 (0.0%) \|  \| \| **SP2_6** \|  \|  \| <0.001 \| \| very much \| 0 (0.0%) \| 4 (8.9%) \|  \| \| quite a lot \| 8 (23.5%) \| 9 (20.0%) \|  \| \| somewhat \| 7 (20.6%) \| 25 (55.6%) \|  \| \| little \| 9 (26.5%) \| 6 (13.3%) \|  \| \| not at all \| 10 (29.4%) \| 1 (2.2%) \|  \| \| **SP3_9** \|  \|  \| 0.001 \| \| very much \| 2 (5.9%) \| 0 (0.0%) \|  \| \| quite a lot \| 4 (11.8%) \| 12 (26.7%) \|  \| \| somewhat \| 13 (38.2%) \| 23 (51.1%) \|  \| \| little \| 4 (11.8%) \| 9 (20.0%) \|  \| \| not at all \| 11 (32.4%) \| 1 (2.2%) \|  \| \| **SP4_12** \|  \|  \| <0.001 \| \| very much \| 1 (2.9%) \| 1 (2.2%) \|  \| \| quite a lot \| 2 (5.9%) \| 6 (13.3%) \|  \| \| somewhat \| 6 (17.6%) \| 24 (53.3%) \|  \| \| little \| 7 (20.6%) \| 13 (28.9%) \|  \| \| not at all \| 18 (52.9%) \| 1 (2.2%) \|  \| \| **SP5_15** \|  \|  \| 0.014 \| \| very much \| 5 (14.7%) \| 4 (8.9%) \|  \| \| quite a lot \| 11 (32.4%) \| 22 (48.9%) \|  \| \| somewhat \| 9 (26.5%) \| 14 (31.1%) \|  \| \| little \| 2 (5.9%) \| 5 (11.1%) \|  \| \| not at all \| 7 (20.6%) \| 0 (0.0%) \|  \| \| **Experienced safety** \|  \|  \| <0.001 \| \| Median (Q1, Q3) \| 12.0 (9.0, 17.0) \| 9.0 (7.0, 11.0) \|  \| \| **ST1_4** \|  \|  \| 0.007 \| \| not at all \| 2 (5.9%) \| 0 (0.0%) \|  \| \| little \| 1 (2.9%) \| 0 (0.0%) \|  \| \| somewhat \| 6 (17.6%) \| 6 (13.3%) \|  \| \| quite a lot \| 13 (38.2%) \| 33 (73.3%) \|  \| \| very much \| 12 (35.3%) \| 6 (13.3%) \|  \| \| **ST2_7** \|  \|  \| 0.062 \| \| little \| 1 (2.9%) \| 1 (2.2%) \|  \| \| somewhat \| 10 (29.4%) \| 10 (22.2%) \|  \| \| quite a lot \| 15 (44.1%) \| 31 (68.9%) \|  \| \| very much \| 8 (23.5%) \| 3 (6.7%) \|  \| \| **ST3_10** \|  \|  \| 0.045 \| \| little \| 10 (29.4%) \| 3 (6.7%) \|  \| \| somewhat \| 7 (20.6%) \| 9 (20.0%) \|  \| \| quite a lot \| 15 (44.1%) \| 26 (57.8%) \|  \| \| very much \| 2 (5.9%) \| 7 (15.6%) \|  \| \| **ST4_13** \|  \|  \| 0.707 \| \| quite a lot \| 1 (2.9%) \| 1 (2.2%) \|  \| \| somewhat \| 7 (20.6%) \| 5 (11.1%) \|  \| \| little \| 12 (35.3%) \| 17 (37.8%) \|  \| \| not at all \| 14 (41.2%) \| 22 (48.9%) \|  \| \| **ST5_16** \|  \|  \| 0.381 \| \| not at all \| 1 (2.9%) \| 0 (0.0%) \|  \| \| little \| 5 (14.7%) \| 2 (4.4%) \|  \| \| somewhat \| 12 (35.3%) \| 16 (35.6%) \|  \| \| quite a lot \| 13 (38.2%) \| 22 (48.9%) \|  \| \| very much \| 3 (8.8%) \| 5 (11.1%) \|  \| \| **Therapeutic hold** \|  \|  \| 0.406 \| \| Median (Q1, Q3) \| 14.0 (11.0, 17.0) \| 14.0 (13.0, 16.0) \|  \| \| **Total EssenCES score** \|  \|  \| 0.003 \| \| Median (Q1, Q3) \| 37.0 (33.0, 43.0) \| 33.0 (30.0, 35.0) \|  \| |

**Supplementary Table 1d: Summary statistics, unit D**

| \|  \| Patient \| Staff \|  \| \| --- \| --- \| --- \| --- \| \|  \| (N = 20) \| (N = 30) \| p-value \| |
| --- | --- | --- | --- | --- | --- | --- | --- | --- |
| \| **CP1_2** \|  \|  \| <0.001 \| \| --- \| --- \| --- \| --- \| \| not at all \| 2 (10.0%) \| 0 (0.0%) \|  \| \| little \| 3 (15.0%) \| 4 (13.3%) \|  \| \| somewhat \| 6 (30.0%) \| 26 (86.7%) \|  \| \| quite a lot \| 8 (40.0%) \| 0 (0.0%) \|  \| \| very much \| 1 (5.0%) \| 0 (0.0%) \|  \| \| **CP2_5** \|  \|  \| 0.017 \| \| not at all \| 5 (25.0%) \| 1 (3.3%) \|  \| \| little \| 2 (10.0%) \| 3 (10.0%) \|  \| \| somewhat \| 7 (35.0%) \| 22 (73.3%) \|  \| \| quite a lot \| 4 (20.0%) \| 4 (13.3%) \|  \| \| very much \| 2 (10.0%) \| 0 (0.0%) \|  \| \| **CP3_8** \|  \|  \| 0.009 \| \| not at all \| 4 (20.0%) \| 0 (0.0%) \|  \| \| little \| 2 (10.0%) \| 4 (13.3%) \|  \| \| somewhat \| 8 (40.0%) \| 23 (76.7%) \|  \| \| quite a lot \| 5 (25.0%) \| 3 (10.0%) \|  \| \| very much \| 1 (5.0%) \| 0 (0.0%) \|  \| \| **CP4_11** \|  \|  \| 0.007 \| \| not at all \| 3 (15.0%) \| 0 (0.0%) \|  \| \| little \| 1 (5.0%) \| 2 (6.7%) \|  \| \| somewhat \| 11 (55.0%) \| 27 (90.0%) \|  \| \| quite a lot \| 4 (20.0%) \| 1 (3.3%) \|  \| \| very much \| 1 (5.0%) \| 0 (0.0%) \|  \| \| **CP5_14** \|  \|  \| 0.014 \| \| not at all \| 2 (10.0%) \| 0 (0.0%) \|  \| \| little \| 0 (0.0%) \| 1 (3.3%) \|  \| \| somewhat \| 12 (60.0%) \| 27 (90.0%) \|  \| \| quite a lot \| 4 (20.0%) \| 2 (6.7%) \|  \| \| very much \| 2 (10.0%) \| 0 (0.0%) \|  \| \| **Patient cohesion** \|  \|  \| 0.464 \| \| Median (Q1, Q3) \| 10.5 (8.0, 12.0) \| 10.0 (9.0, 10.0) \|  \| \| **SP1_3** \|  \|  \| 0.003 \| \| very much \| 5 (25.0%) \| 14 (46.7%) \|  \| \| quite a lot \| 1 (5.0%) \| 10 (33.3%) \|  \| \| somewhat \| 10 (50.0%) \| 4 (13.3%) \|  \| \| little \| 3 (15.0%) \| 2 (6.7%) \|  \| \| not at all \| 1 (5.0%) \| 0 (0.0%) \|  \| \| **SP2_6** \|  \|  \| 0.047 \| \| very much \| 4 (20.0%) \| 12 (40.0%) \|  \| \| quite a lot \| 3 (15.0%) \| 9 (30.0%) \|  \| \| somewhat \| 6 (30.0%) \| 7 (23.3%) \|  \| \| little \| 3 (15.0%) \| 2 (6.7%) \|  \| \| not at all \| 4 (20.0%) \| 0 (0.0%) \|  \| \| **SP3_9** \|  \|  \| 0.006 \| \| very much \| 2 (10.0%) \| 4 (13.3%) \|  \| \| quite a lot \| 3 (15.0%) \| 14 (46.7%) \|  \| \| somewhat \| 7 (35.0%) \| 11 (36.7%) \|  \| \| little \| 3 (15.0%) \| 1 (3.3%) \|  \| \| not at all \| 5 (25.0%) \| 0 (0.0%) \|  \| \| **SP4_12** \|  \|  \| <0.001 \| \| very much \| 1 (5.0%) \| 3 (10.0%) \|  \| \| quite a lot \| 2 (10.0%) \| 9 (30.0%) \|  \| \| somewhat \| 2 (10.0%) \| 13 (43.3%) \|  \| \| little \| 2 (10.0%) \| 3 (10.0%) \|  \| \| not at all \| 13 (65.0%) \| 2 (6.7%) \|  \| \| **SP5_15** \|  \|  \| <0.001 \| \| very much \| 5 (25.0%) \| 9 (30.0%) \|  \| \| quite a lot \| 1 (5.0%) \| 17 (56.7%) \|  \| \| somewhat \| 8 (40.0%) \| 3 (10.0%) \|  \| \| little \| 4 (20.0%) \| 1 (3.3%) \|  \| \| not at all \| 2 (10.0%) \| 0 (0.0%) \|  \| \| **Experienced safety** \|  \|  \| <0.001 \| \| Median (Q1, Q3) \| 11.5 (8.5, 15.0) \| 6.0 (3.0, 8.0) \|  \| \| **ST1_4** \|  \|  \| 0.002 \| \| not at all \| 2 (10.0%) \| 0 (0.0%) \|  \| \| little \| 3 (15.0%) \| 0 (0.0%) \|  \| \| somewhat \| 5 (25.0%) \| 1 (3.3%) \|  \| \| quite a lot \| 5 (25.0%) \| 18 (60.0%) \|  \| \| very much \| 5 (25.0%) \| 11 (36.7%) \|  \| \| **ST2_7** \|  \|  \| 0.066 \| \| not at all \| 3 (15.0%) \| 1 (3.3%) \|  \| \| little \| 1 (5.0%) \| 0 (0.0%) \|  \| \| somewhat \| 4 (20.0%) \| 3 (10.0%) \|  \| \| quite a lot \| 9 (45.0%) \| 12 (40.0%) \|  \| \| very much \| 3 (15.0%) \| 14 (46.7%) \|  \| \| **ST3_10** \|  \|  \| 0.148 \| \| not at all \| 3 (15.0%) \| 0 (0.0%) \|  \| \| little \| 2 (10.0%) \| 1 (3.3%) \|  \| \| somewhat \| 3 (15.0%) \| 3 (10.0%) \|  \| \| quite a lot \| 7 (35.0%) \| 15 (50.0%) \|  \| \| very much \| 5 (25.0%) \| 11 (36.7%) \|  \| \| **ST4_13** \|  \|  \| 0.003 \| \| very much \| 3 (15.0%) \| 0 (0.0%) \|  \| \| quite a lot \| 1 (5.0%) \| 1 (3.3%) \|  \| \| somewhat \| 2 (10.0%) \| 4 (13.3%) \|  \| \| little \| 7 (35.0%) \| 2 (6.7%) \|  \| \| not at all \| 7 (35.0%) \| 23 (76.7%) \|  \| \| **ST5_16** \|  \|  \| 0.035 \| \| not at all \| 2 (10.0%) \| 0 (0.0%) \|  \| \| little \| 5 (25.0%) \| 1 (3.3%) \|  \| \| somewhat \| 4 (20.0%) \| 13 (43.3%) \|  \| \| quite a lot \| 7 (35.0%) \| 10 (33.3%) \|  \| \| very much \| 2 (10.0%) \| 6 (20.0%) \|  \| \| **Therapeutic hold** \|  \|  \| 0.005 \| \| Median (Q1, Q3) \| 14.0 (9.5, 16.0) \| 16.5 (14.0, 18.0) \|  \| \| **Total EssenCES score** \|  \|  \| 0.198 \| \| Median (Q1, Q3) \| 36.0 (27.5, 40.0) \| 31.5 (28.0, 35.0) \|  \| |

**Supplementary Table 2: Factor loadings following principal components analysis using orthogonal varimax rotation**

|  | **Component** |  |  |
| --- | --- | --- | --- |
|  | **Factor 1** | **Factor 2** | **Factor 3** |
| **Item** | **Experienced safety** | **Patient cohesion** | **Therapeutic hold** |
| CP1 |  | 0.63 |  |
| CP2 |  | 0.58 |  |
| CP3 |  | 0.69 |  |
| CP4 |  | 0.64 |  |
| CP5 |  | 0.62 |  |
| SP1 | 0.76 |  |  |
| SP2 | 0.82 |  |  |
| SP3 | 0.75 |  |  |
| SP4 | 0.62 |  |  |
| SP5 | 0.77 |  |  |
| ST1 |  |  | 0.65 |
| ST2 |  |  | 0.57 |
| ST3 |  |  | 0.74 |
| ST4 |  |  | 0.63 |
| ST5 |  |  | 0.38 |
